# Gray matter atrophy in relapsing-remitting multiple sclerosis is associated with white matter lesions in connecting fibers

**DOI:** 10.1177/13524585211044957

**Published:** 2021-09-30

**Authors:** Matthias Bussas, Sophia Grahl, Viola Pongratz, Achim Berthele, Christiane Gasperi, Till Andlauer, Christian Gaser, Jan S Kirschke, Benedikt Wiestler, Claus Zimmer, Bernhard Hemmer, Mark Mühlau

**Affiliations:** Department of Neurology, School of Medicine, Technical University of Munich, Munich, Germany/TUM-Neuroimaging Center, School of Medicine, Technical University of Munich, Munich, Germany; Department of Neurology, School of Medicine, Technical University of Munich, Munich, Germany/TUM-Neuroimaging Center, School of Medicine, Technical University of Munich, Munich, Germany; Department of Neurology, School of Medicine, Technical University of Munich, Munich, Germany/TUM-Neuroimaging Center, School of Medicine, Technical University of Munich, Munich, Germany; Department of Neurology, School of Medicine, Technical University of Munich, Munich, Germany; Department of Neurology, School of Medicine, Technical University of Munich, Munich, Germany; Department of Neurology, School of Medicine, Technical University of Munich, Munich, Germany; Department of Psychiatry and Department of Neurology, Jena University Hospital, Jena, Germany; Department of Neuroradiology, School of Medicine, Technical University of Munich, Munich, Germany; Department of Neuroradiology, School of Medicine, Technical University of Munich, Munich, Germany; Department of Neuroradiology, School of Medicine, Technical University of Munich, Munich, Germany; Department of Neurology, School of Medicine, Technical University of Munich, Munich, Germany/Munich Cluster for Systems Neurology (SyNergy), Munich, Germany; Department of Neurology, School of Medicine, Technical University of Munich, Munich, Germany/TUM-Neuroimaging Center, School of Medicine, Technical University of Munich, Munich, Germany

**Keywords:** Biomarkers, multiple sclerosis, outcome measurement, relapsing/remitting, T2 lesions, atrophy

## Abstract

**Background:**

Lesions of brain white matter (WM) and atrophy of brain gray matter (GM) are well-established surrogate parameters in multiple sclerosis (MS), but it is unclear how closely these parameters relate to each other.

**Objective:**

To assess across the whole cerebrum whether GM atrophy can be explained by lesions in connecting WM tracts.

**Methods:**

GM images of 600 patients with relapsing-remitting MS (women = 68%; median age = 33.0 years, median expanded disability status scale score = 1.5) were converted to atrophy maps by data from a healthy control cohort. An atlas of WM tracts from the Human Connectome Project and individual lesion maps were merged to identify potentially disconnected GM regions, leading to individual disconnectome maps. Across the whole cerebrum, GM atrophy and potentially disconnected GM were tested for association both cross-sectionally and longitudinally.

**Results:**

We found highly significant correlations between disconnection and atrophy across most of the cerebrum. Longitudinal analysis demonstrated a close temporal relation of WM lesion formation and GM atrophy in connecting fibers.

**Conclusion:**

GM atrophy is associated with WM lesions in connecting fibers. Caution is warranted when interpreting group differences in GM atrophy exclusively as differences in early neurodegeneration independent of WM lesion formation.

## Introduction

Multiple sclerosis (MS) is an autoimmune inflammatory disease of the central nervous system (CNS). Demyelinating lesions in CNS white matter (WM) are the hallmark of MS. In most instances, the earlier stage of MS is coined by unpredictable episodes of neurological deficits because of new WM lesions resulting from acute inflammation (relapsing-remitting multiple sclerosis, RRMS). On T2-weighted magnetic resonance images (MRIs), demyelinating WM lesions are hyperintense. Their detection has become the paraclinical mainstay to diagnose MS and to monitor its course.^1^ The phase of RRMS is usually followed by a gradual accumulation of neurological deficits independent of demyelinating attacks, albeit at highly variable intervals (secondary progressive MS). This later stage of MS is less well-understood with neurodegenerative processes coming more and more into play. It is remarkable in this context that MS-related tissue damage by various pathological processes, beyond demyelinating WM lesions, has been described in virtually all compartments of the CNS.^2^ These processes are assumed to contribute to neurodegeneration and, hence, to brain atrophy, which is first most pronounced in gray matter (GM).^3^ Accordingly, WM lesions and brain atrophy are the two most established MRI-based parameters in MS research and have served as surrogates in clinical trials.^4^ This has further been justified by demonstrating that, although correlated,^5^ each of the two global measures of brain pathology independently explains variance of MS-related disability.^6^ In the stage of RRMS, brain GM atrophy is commonly seen as a summary measure of MS-related tissue damage complementary to WM lesions, namely, early neurodegeneration eventually leading to, or at least contributing to, secondary progression.^4^ Alternatively, WM lesions may drive brain atrophy directly through axonal damage of lesions in connecting WM fibers. In this case, both measures would not reflect different aspects of MS-related pathology. This would also challenge the idea of differential meanings for later individual disease courses. Instead, both measures would reflect two sides of the same coin, namely, the direct appearance and the systemic consequences of WM lesion formation. This is also well conceivable since MS-related WM lesions contain transected axons,^7^ and it has been known for long that transection of axons leads to atrophy of connected GM areas.^8^ Given the many lines of evidence for both mechanisms, they are likely to coexist.

Here, we investigate whether WM lesions in connecting fibers contribute to GM atrophy as measurable by brain MRI. This requires precise knowledge on the spatial course of WM fibers. This precise knowledge on the spatial course of brain WM tracts has become available recently particularly through the Human Connectome Project.^9^ Harnessing this knowledge has been regarded key to better understand the consequences of brain lesions across the variety of neuropsychiatric disorders.^10^ We followed this idea and applied a newly developed method in a large cohort of patients in the stage of RRMS. In our analyses, we related maps of GM atrophy to maps of potential disconnection through WM lesions; this way, we investigated whether GM atrophy can be explained by WM lesion location and, hence, most likely through axonal damage by lesions in connecting WM fibers.

## Methods

### Participants

The study was performed in accord with the Declaration of Helsinki. Patients had given informed consent to the use of their data for research purposes. The study was approved by the ethics committee of the School of Medicine, Technical University of Munich, Germany. Data from healthy controls, who had participated in other imaging studies at our institution, were derived from our in-house database. We considered data of all patients included in our in-house cohort study on MS (TUM-MS) of the Department of Neurology, Klinikum Rechts der Isar, Munich, Germany. In the context of TUM-MS, we have followed a large cohort of patients with an established diagnosis of clinically isolated syndrome or MS. The follow-up scheme includes yearly brain MRI scans. All MRI scans were acquired with the same standardized protocol at the same scanner, which was exclusively used between 1 January 2009 and 30 November 2017. Inclusion criteria were availability of at least two standardized MRI with an interscan interval of at least 6 months, a diagnosis of RRMS according to the current diagnostic criteria^1^ at the first time point. When images of multiple time points were available, we used the first one for cross-sectional analyses. For the longitudinal analyses, we chose the first and last scan in case of more than two time points.

### MRI acquisition and processing

High-resolution MRI was performed at the 3 Tesla scanner Achieva (Philips Medical Systems, Netherlands). Three-dimensional (3D) spoiled gradient echo T1-weighted (T1w) sequences were applied with the following parameters: voxel size = 1 mm isotropic; TR = 9 ms; TE = 4 ms. Furthermore, turbo-spin echo T2-weighted fluid-attenuated inversion recovery (FLAIR) images were acquired with the following parameters: voxel size = 1.0 mm × 1.0 mm × 1.5 mm; TR = 10,000 ms; TE = 140 ms; TI = 2750 ms.

The images were processed with the software package SPM12 (http://www.fil.ion.ucl.ac.uk/spm/software/spm12/) and its toolboxes CAT12 (http://www.neuro.uni-jena.de/cat/index.html) and LST (lesion segmentation tool) 2.0.15 (http://statistical-modeling.de/lst.html). The T1w and FLAIR images were denoised using the denoising filter (Spatial Adaptive Non-Local Means Denoising) of CAT.^11^ WM lesion segmentation was performed using the lesion growth algorithm of LST.^12^ Based on the resulting WM lesion maps, the lesion voxels in the T1w images were removed and filled with normal-appearing WM using the lesion-filling algorithm of LST. The filled T1w images were warped (normalized) into the standard brain space of the Montreal Neurological Institute (MNI) and segmented into GM, WM, and cerebrospinal fluid using CAT. All images were transformed into a 2-mm isotropic MNI space for compatibility with the connectome data. We also used CAT to compute surface-based maps of cortical thickness.^13^

### Individual atrophy maps

To capture the atrophy of the MS cohort, we applied the following process. Based on the GM images of 131 healthy controls (94 women, mean age ± standard deviation = 32.0 ± 8.7 years, median = 30, range = 18–60), we estimated a linear model in analogy to the Template-O-Matic toolbox.^14^ This model predicted individual GM images from the variables sex, age, and total intracranial volume. Using this model, we estimated GM atrophy maps of all patients by calculating the difference between the predicted and the true GM image. By this procedure, we obtained voxel- and surface-based atrophy maps.

### Connectome data

The connectome^15^ was rendered from 842 healthy subjects from data of the Human Connectome Project.^9^ This “Yeh connectome” is based on whole brain tractography and the result was cleaned and enriched by expert knowledge—leading to 550,000 fibers.

### Computation of disconnectome maps

**Figure 1..**
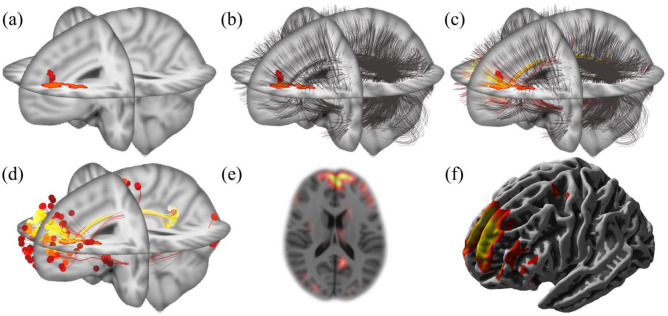
Computation of an individual disconnectome map. (a) Lesion map. A binary map of white matter lesions, derived from T1-weighted and FLAIR images, was warped (normalized) to the standard brain space of the Montreal Neurological Institute (MNI space). (b) Coregistration of the connectome. The white matter fibers (black stripes) derived from the connectome template (provided in MNI space) are overlaid on the binary white matter lesion map. (c) Scoring of fibers. White matter fibers are scored according to their overlap with white matter lesions. This is color-coded from red to yellow corresponding to the degree of overlap from low to high. (d) Score projection to fiber endpoints. Summary scores of white matter lesion overlap are projected to fiber endpoints. (e) This can be done in a voxel-wise manner, as illustrated by an axial slice, and (f) in a surface-based manner, as illustrated by a lateral view.

The computation of the individual disconnectome map (Figure 1) starts with overlaying the normalized WM lesion map (Figure 1(a)) and the connectome data (Figure 1(b)). We then iterate over all fibers of the connectome and assign a score to each of them as follows. For each individual fiber, we first detect all voxels that it passes through using Bresenham’s algorithm^16^ and then count those voxels that are part of a lesion (Figure 1(c)). We then propagate this lesion voxel count into the two endpoints of the fiber (Figure 1(d)). After mapping these endpoints back into voxel space, the individual disconnectome is computed as the sum of all these scored endpoints. Finally, we smooth these maps using an 8-mm Gaussian kernel and mask the image to only contain disconnection scores in the GM using the GM template of SPM12 (Figure 1(e)). We also generate a surface-based version of the disconnectome using the surface tools of CAT to enable analyses focusing on cortical atrophy through determination of cortical thickness (Figure 1(f)).

As all steps in this computation (including smoothing and masking) are linear, we can precompute the disconnectome for every possible lesion voxel individually. The final disconnectome is the sum of these individual disconnectomes for all lesion voxels in a lesion map. This procedure enables the computation of disconnectomes within seconds.

### Statistical analysis

To ensure whole cerebrum GM coverage of the disconnectome template, we computed a map of endpoints of the raw connectome templates and assessed whether they reach all GM areas. We also computed mean voxel- and surface-based atrophy maps to locate areas with the highest degree of potential disconnection through WM lesions. To study the relation between regional GM atrophy and potential GM disconnection cross-sectionally, disconnectome maps of the first time points were correlated with their respective atrophy maps by calculating Pearson’s correlation coefficients in a voxel-wise manner. Likewise, we performed a vertex-wise correlation analysis between the surface-based disconnectome maps and the cortical atrophy maps. GM regions, identified by the voxel-wise analysis, were localized by the atlas of Hammers^17,18^ as implemented in CAT12. For each region, maximum *r*-values were extracted. To demonstrate that our approach explains more variance of GM atrophy than a simple voxel-wise or surface-based correlation of GM atrophy with overall WM lesion volume, we repeated both analyses, each extended by the control variable of WM lesion volume. For the longitudinal analysis, scans of both time points of each patient were analyzed. WM lesion increase was calculated by subtracting WM lesion maps of time point 1 from their individual counterparts of time point 2; these maps of WM lesion increase were used to calculate maps of disconnection increase (as described above). Maps of GM atrophy increase were calculated by subtracting GM atrophy maps of time point 1 from their respective counterparts of time point 2. Maps of disconnection increase were correlated with their respective maps of atrophy increase. Again, we conducted this experiment voxel-wise and surface-based and computed an additional version correcting for the increase in WM lesion volume. All voxel- and surface-based maps of *p*-values were False Discovery Rate (FDR) corrected and only FDR levels of less than 5% were considered significant.

## Results

### Study participants

From our database, we identified 614 patients with an established diagnosis of RRMS and at least two available standardized brain MRI. We excluded one patient as our image processing pipeline could not process these images. All images were visually inspected. After inspection of the raw images, seven patients were excluded because of MRI artifacts (three patients) or findings likely to interfere with image processing such as arachnoid cysts (four patients); after inspection of the processed images, another six patients were excluded because of poor processing results (insufficient lesion segmentation in three patients and unsatisfactory lesion filling in another three patients). Finally, the cohort available for analysis comprised 600 patients. Key characteristics of this cohort are summarized in Table 1.

**Table 1..** Key characteristics of the cohort at the first time point.

*N*	600
Age in years	34.5 ± 9.9; 33.0 [18.0–66.5]
Females (in %)	405 (67.5%)
EDSS	1.4 ± 1.1; 1.5 [0–6.5]
Disease duration in years	1.5 ± 3.6; 0.3 [0–13.8]
Time between scans in years	4.0 ± 2.2; 4.0 [0.5–8.8]
Volume of white matter lesions (mL)	4.8 ± 8.0; 2.1 [0–77]
Volume of new white matter lesions (mL) between first and second time points	2.7 ± 4.6; 1.1 [0–44]
Disease-modifying therapies	
None	419
Aza/DMF/FTY/Glat/INF/NTZ/Terifl	1/10/8/28/105/28/1

Aza: azathioprine; DMF: dimethyl fumarate; EDSS: Expanded Disability Status Scale; FTY: fingolimod; Glat: glatiramer acetate; INF: interferon-β, NTZ: natalizumab; Terifl: teriflunomide.

None indicates no disease-modifying therapy.

Values are given in mean value ± standard deviation and in median [range].

### Coverage of the cerebral GM by the connectome template and degree of GM atrophy

**Figure 2..**
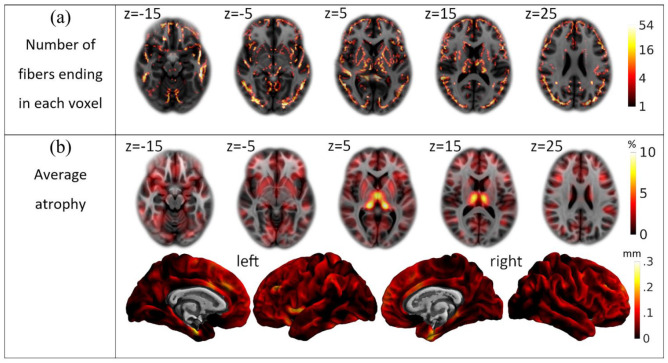
Descriptive statistics. (a) Whole brain coverage by the Yeh connectome. The number of fibers ending in each voxel is projected onto the standard brain and color-coded according to a logarithmic scale ranging from 1 to 54 as indicated by the bar on the right. (b) Average atrophy. The mean atrophy map of the cross-sectional cohort is projected onto the standard brain space of the Montreal Neurological Institute and color-coded according to the bars on the right (top, voxel-wise images in percent of gray matter loss; bottom, surface-based images in mm of cortical thickness decrease).

The map indicating the number of fiber endings of the connectome template across the whole cerebrum demonstrated a widespread coverage of GM areas although with varying intensity (Figure 2(a)). To estimate the amount of MS-related atrophy across the cohort, we computed the average of the atrophy maps of the 600 patients in a voxel-wise and surface-based manner (Figure 2(b)). The voxel-wise average showed atrophy in all GM areas of at least 2.5%. Deep GM areas, most prominently the thalamus, showed the strongest atrophy of up to 10%. The surface-based results showed a relatively uniform cortical thinning of around 0.1 mm. This effect was most prominent in both medial temporal lobes, where the thinning increased to 0.3 mm.

### Cross-sectional correlation of GM atrophy and disconnection

**Figure 3..**
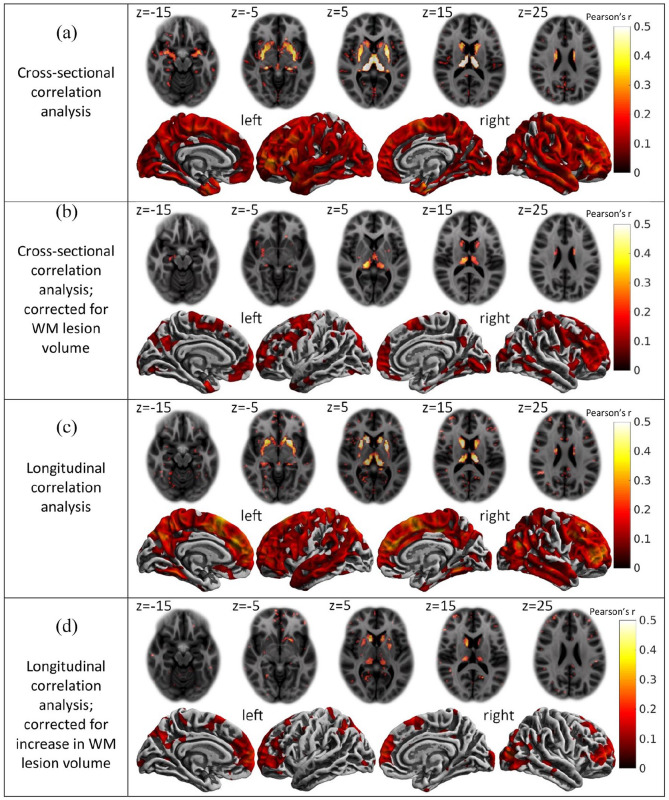
Correlation of disconnection with gray matter atrophy. (a) Cross-sectional correlations between disconnectome maps and gray matter atrophy maps. (b) Cross-sectional correlations extended by the control variable of white matter (WM) lesion volume. (c) Longitudinal correlations between maps of disconnection increase and maps of atrophy increase. (d) Longitudinal correlations between maps of disconnection increase and maps of atrophy increase extended by the control variable of the volume of new white matter (WM) lesions. (a)–(d) Each panel contains axial slices of the voxel-wise correlations (top) as well as medial and lateral views of the surface-based correlations (bottom). *Z*-coordinates of the standard brain space of the Montreal Neurological Institute are indicated for axial slices. The colors indicate the strength of the correlation according to the bars on the right (Pearson’s *r*). Only voxels or vertices that were significant on a 5% false discovery rate level are colored.

We found a highly significant, strong, and widespread correlation of GM atrophy and disconnection (Figure 3(a) and Table 2). The voxel-wise analysis showed a strong positive correlation of GM atrophy and disconnection in deep GM and a widespread positive correlation in the cortex. Pearson’s *r* in the thalami averaged around 0.65 (*p*-value < 10^−16^), and in the putamen and caudate, the values were between 0.35 and 0.5 (*p* < 10^−16^); the correlation in the cortex peaked with values around 0.25 (*p* < 10^−9^). The surface-based analysis showed a relatively uniform positive correlation with Pearson’s *r* between 0.2 and 0.4 (*p* < 10^−9^). After extension of the model by the control variable of WM lesion volume, the correlation of GM atrophy and disconnection remained significant in widespread areas of deep and cortical GM (Figure 3(b) and Table 2).

**Table 2..** Correlations of disconnectome maps with gray matter atrophy across cerebral regions.

Region	*r*-value	Region	*r*-value	Region	*r*-value
Frontal		Deep GM/insula		Temporal	
Ant. Cinguli G.	0.21/0.12	Insula	0.47/0.20	Hippocampus	0.36/0.15
Precentral G.	0.25/0.20	Accumbens Nucleus	0.24/ns	Amygdala	0.32/0.13
Superior Frontal G.	0.18/0.15	Caudate Nucleus	0.46/0.33	Ambient and Parahippocampus G.	0.26/0.14
Middle Frontal G.	0.34/0.30	Putamen	0.52/0.25	Ant. Medial Temp. Lobe	0.34/0.18
Inf. Frontal G.	0.22/0.16	Pallidum	0.37/0.16	Ant. Lateral Temp. Lobe	0.22/0.16
G. Rectus	0.19/0.13	Thalamus	0.74/0.40	Superior Temp. G.	0.26/0.14
Orbito-Frontal G.	0.22/0.16	Parietal		Inf. Middle Temp. G.	0.20/0.17
Occipital		Posterior Cinguli G.	0.21/0.13	Fusiform G.	0.19/0.14
Lateral Occipital Lobe	0.23/0.16	Postcentral G.	0.21/0.17	Posterior Temp. Lobe	0.51/0.18
Lingual G.	0.24/0.13	Superior Par. G.	0.24/0.15		
Cuneus	0.21/0.13	Inf. Lateral Par. Lobe	0.22/0.16		

Ant.: anterior; Inf..: inferior; ns: not significant; Par., parietal; Temp., temporal; G.: gyrus.

Regions were taken from the atlas by Hammers and colleagues.^17,18^ Maximum *r*-values of each region from voxel-wise analyses without/with correction for white matter lesion volume (corresponding to Figure 3(a) and (b)) are indicated. All maximum values went along with corrected *p*-values < 0.01. No striking left/right differences were observed and values from the left and right hemispheres merged.

### Longitudinal correlation analysis of GM atrophy and disconnection

We also observed a strong correlation between the increases in disconnection with the increases in GM atrophy (Figure 3(c)). The voxel-wise analysis revealed a strong correlation in the deep GM with Pearson’s *r*-values between 0.2 and 0.5 (*p* < 10^−6^) reaching maximum values in the thalamus. The surface-based analysis showed a widespread correlation of the cerebral cortex with Pearson’s *r*-values up to 0.4 (*p* < 10^−16^). After extension of the model by the control variable of new WM lesion volume (Figure 3(d)), the correlation of GM atrophy and disconnection remained significant primarily in areas with the largest effect sizes in the model without correction for WM lesion volume increase (Figure 3(c)).

## Discussion

In this large cohort of patients with RRMS, we holistically show that cerebral GM atrophy is associated with WM lesions in connecting fibers. We regard damage within WM lesions, leading to subsequent degeneration along axonal projections, the most likely underlying mechanism.

We did not expect a simple “yes” or “no” answer to the question whether lesions in projecting WM fibers contribute to regional GM atrophy. On one hand, we were aware of the many lines of evidence for direct MS-related tissue damage in GM.^3^ On the other hand, there is also much evidence for remote consequences of WM lesions and changes in normal-appearing WM resulting in GM atrophy as measurable by structural MRI. Such a relation was demonstrated for thalamic regions,^19–21^ deep GM,^22^ the visual system,^23^ the corticospinal tract,^24,25^ and a selection of deep and cortical GM regions.^26,27^ Further approaches revealed a relation of WM abnormality with connected GM,^28–30^ and it could be shown that spatial patterns of GM atrophy are not random but occur in distinct spatial pattern; of note, these patterns were associated with WM lesion load, as modeled in this study, but only to a lesser extent with decreases in normal-appearing WM integrity.^31^ Moreover, an association of overall disruption of WM tracts and clinical symptoms could be demonstrated in MS.^32–34^ In consequence, we aimed to demonstrate one of the two categories of GM damage, namely, disconnection by WM lesions. More precisely, we aimed to demonstrate this mechanism of GM atrophy holistically (i.e. across the whole cerebrum) through more recently available knowledge on WM tract anatomy, to better understand the relation of GM atrophy and WM lesion load, as given as summary measures for the whole brain in clinical trials.

We made use of a template on WM tract anatomy.^15^ Of note, the first such template, we are aware of, was based on data from a comparatively small cohort of 169 subjects and still validated by comparison with the gold standard technique for the in vivo investigation of WM tracts in humans, diffusion tensor imaging.^35^ The template used here is based on data from a large cohort of the Human Connectome Project.^9^ Besides careful and extensive visual analysis of disconnectome maps derived from patients with only few lesions in WM tracts with well-known projections in advance of the study, we derived a map with an overlay of all ends of fibers of the WM tract template to ensure full coverage of cerebral GM (Figure 2(a)). In addition, we estimated individual GM atrophy; this way we could approximate the variance of MS-related atrophy explainable by WM lesions in connecting fibers across our cohort. Our atrophy maps were an approximation, accounting for age, sex and total intracranial volume, but not fully specific for MS-related atrophy; instead, it also entails physiological variance which most likely decreases the degree of GM atrophy explainable by our model. Furthermore, we had to decide how to measure GM. Two main categories of processing techniques are available, namely, voxel-based and surface-based techniques. There is no common agreement on the superiority of one technique over the other. In MS, a good agreement between GM content derived from a voxel-based method and histologically determined neuronal density, neuronal size, and axonal density was demonstrated;^36^ likewise, a good agreement was found between cortical thickness derived from a surface-based method and cortical thickness derived histologically.^37^ In addition, full coverage of deep GM is only provided by voxel-based methods. We therefore decided to use both a voxel-based method and a surface-based method. We chose a software package containing both methods. This software (SPM12/CAT12) is well-established for voxel-based morphometry and evidence for a reliable determination of cortical thickness has been provided^38^ also in MS.^39^

In our cross-sectional analyses, we found a strong and highly significant association of regional GM atrophy with WM lesions in connecting fibers. Of note, this relation remained significant in widespread areas of cerebral GM after extending the statistical model by the control variable of WM lesion volume. Hence, our technique seems to be able to explain GM atrophy better than the standard parameter of overall WM lesion volume. Furthermore, the pattern of correlation strengths across cerebral GM (anterior > posterior regions) resembled that of observed atrophy (Figures 3(b) and 2(b)). This might indicate that smaller observed correlations result from reduced statistical power due to less explainable variance rather than a weaker mechanism of disconnection. By longitudinal analyses, we tested whether the formation of WM lesions is in close temporal association with the increase in GM atrophy in connecting regions. Compatible with our hypothesis, we observed a striking correlation of both measures. Of note, these correlations differed between GM regions being more pronounced in deep GM than in cortical GM. After extension of this model by the control variable of the volume of new WM lesions, the spatial extent of areas with a significant correlation of GM atrophy and disconnection decreased. We assume that this may result from a loss of statistical power, since significance remained primarily in areas with the largest effect sizes in the longitudinal model without correction for WM lesion volume.

We acknowledge limitations of this study. Our cohort consisted of patients with RRMS so that we can neither conclude on the later phase of secondary progressive MS nor on primary progressive MS. The applied WM fiber template and hence our analyses did neither cover the infratentorial compartment nor the spinal cord. That is why we refer to cerebrum instead of brain. We did not model possible higher order systemic effects.^40^ With conventional MRI sequences, we could neither directly measure the degree of axonal transection within a given WM lesion volume nor identify the exact course of individual WM tracts. Although individual images were normalized, the use of a single WM fiber template may be a relatively rough approximation. GM regions of potential disconnection were inferred from fiber ends of the template which may have led to imperfect coregistration. Furthermore, our approach treats all WM lesions the same way (given that they were recognized as hyperintense on our FLAIR sequence). Lesion voxels along the same tract are seen as additional disconnection. This is more intuitive for demyelination, where the effect may be additive, than for axonal transection. The smoothing kernel of 8 mm is well-established in the field of structural neuroimaging but may not have been optimal for our analyses. Lesion segmentation was performed by LST which tends to miss few, mostly small, lesions particularly near the cortex. In consequence, our method is based on approximations most likely resulting in a decrease of explainable variance. Yet, compared to effect sizes typically reported in the field of neuroimaging, explained variance was high, particularly for deep GM atrophy with almost 50%. Still, up to 50% of variance is left unexplained so that it remains a matter of perspective to pronounce one or the other aspect.^27^

We conclude that, in RRMS, GM atrophy is associated with WM lesions in connecting fibers, although the strength of the relationship varied substantially from region to region. Caution is warranted when interpreting group differences in GM atrophy, including those reported in clinical trials, exclusively as differences in early neurodegeneration independent of WM lesion formation.
